# Surface Functionalization of Polyethylene Granules by Treatment with Low-Pressure Air Plasma

**DOI:** 10.3390/ma11060885

**Published:** 2018-05-25

**Authors:** Hana Šourková, Gregor Primc, Petr Špatenka

**Affiliations:** 1 Faculty of Mechanical Engineering, Department of Materials Engineering, Center of Advanced Aerospace Technology, Czech Technical University in Prague, Karlovo náměstí 13, 121 35 Praha 2, Czech Republic; hana.sourkova@tul.cz (H.Š.); gregor.primc@ijs.si (G.P.); 2 Faculty of Mechatronics, Informatics and Interdisciplinary Studies, Technical University of Liberec, Studentská 1402/2, 461 17 Liberec 1, Czech Republic; 3 Jozef Stefan Institute, Jamova cesta 39, 1000 Ljubljana, Slovenia

**Keywords:** polyethylene granules, low-pressure MW air plasma, optical emission spectroscopy, XPS, laser cobalt catalytic probe

## Abstract

Polyethylene granules of diameter 2 mm were treated with a low-pressure weakly ionized air plasma created in a metallic chamber by a pulsed microwave discharge of pulse duration 180 μs and duty cycle 70%. Optical emission spectroscopy showed rich bands of neutral nitrogen molecules and weak O-atom transitions, but the emission from N atoms was below the detection limit. The density of O atoms in the plasma above the samples was measured with a cobalt catalytic probe and exhibited a broad peak at the pressure of 80 Pa, where it was about 2.3 × 10^21^ m^−3^. The samples were characterized by X-ray photoelectron spectroscopy. Survey spectra showed oxygen on the surface, while the nitrogen concentration remained below the detection limit for all conditions. The high-resolution C1s peaks revealed formation of various functional groups rather independently from treatment parameters. The results were explained by extensive dissociation of oxygen molecules in the gaseous plasma and negligible flux of N atoms on the polymer surface.

## 1. Introduction

Surface properties of polymers are often inadequate, so they have to be modified prior to further treatment. A widely used technique for tailoring the surface properties of polymers is a brief exposure to non-equilibrium gaseous plasma. A recent survey indicates a variety of treatment conditions, and different authors reported results that are not always in agreement [1]. A possible reason for such discrepancies is the application of polymers in various forms and various grades. Recently, it was explained in detail that fibrous polymers might interact differently with gaseous plasma compared to interaction with flat materials, such as foils, due to the enormous surface-to-mass ratio [2]. Although numerous studies have been published in past decades, the exact mechanisms are still not well understood, so the interaction of plasma particles with polymer surfaces remains a hot topic [3]. A commonly used polymer is polyethylene, which comes in different forms and different grades. Recently, Orendač et al. studied the modification of high-density polyethylene (HDPE) foils by argon plasma to create allyl and polyenyl radicals or dangling bonds [4]. Vartiainen et al. [5] applied gaseous plasma for the activation of low-density polyethylene, prior to coating it with a thin film of cellulose nanofibrils to make an effective barrier against oxygen and mineral oil residues. Zhao et al. [6] treated polypropylene fibrous membranes with weakly ionized plasma to activate the surface for better adhesion of a coating prepared by grafting with vinylimidazole acidic ionic liquids. Hu et al. treated a polyethylene surface with CO_2_ plasma for carbene insertion [7]. Ozaltin et al. [8] used plasma pretreatment of low-density polyethylene (LDPE) before grafting of polymer brush of *N*-allylmethylamine, which acted as an antibacterial coating. Muzammil et al. [9] used a pulsed plasma technique for the deposition of hydrophobic coatings on low-density polyethylene. Van Vrekhem et al. [10] used an atmospheric pressure plasma jet for the treatment of ultrahigh-density polyethylene in order to improve the biological response of shoulder implants, and found improved adhesion to bone cement and an enhanced osteoblast proliferation. Lindner et al. [11] used a high-impedance corona discharge in air for the treatment of various polymers and laminates, and the best results in terms of laminate bond strength were obtained for low-density polyethylene. Popelka et al. [12] used corona discharge to enhance the surface hydrophilicity of composites prepared from linear low-density polyethylene and graphene nanoplatelets in order to develop materials suitable for electromagnetic interference shielding applications. The same group also applied the same discharge to obtain a significant increase in peel resistance in the linear low-density polyethylene/aluminum laminate [13]. This literature survey highlights important achievements reported in papers published only in 2018. Much work on polyethylene in different forms has been done in previous years using low-pressure microwave plasma: from the functionalization of low-density polypropylene with nitrogen plasma [14] and studies on the wetting properties of LDPE and polyethylene terephthalate (PET) [15] to the enhanced printability of polyethylene (PE) treated by air plasma [16]. Besides PE granules and foils, work has been also done on powders [17,18,19]. The complete survey is beyond the scope of this paper, but a general conclusion is that sometimes even a brief treatment of polyethylene causes improved surface wettability, which is a consequence of polar functional groups that appear on the surface upon plasma treatment. Usually, the surface is rich in oxygen-containing functional groups such as hydroxyl, carbonyl, and carboxyl/ester, even though the plasma was not generated in pure oxygen, but rather air, carbon dioxide, or even argon. The richness of oxygen-containing groups is usually explained by an excellent affinity of oxygen towards plasma-modified polymer surface. The authors used polymers in different forms, including foils and fibrous materials, but little work has been performed on the treatment of granules, which are the raw materials for synthesizing a variety of plastics. This paper reports on the surface modification of polyethylene granules treated with air plasma sustained in a chamber suitable for semi-industrial applications.

## 2. Materials and Methods

**Figure 1:**
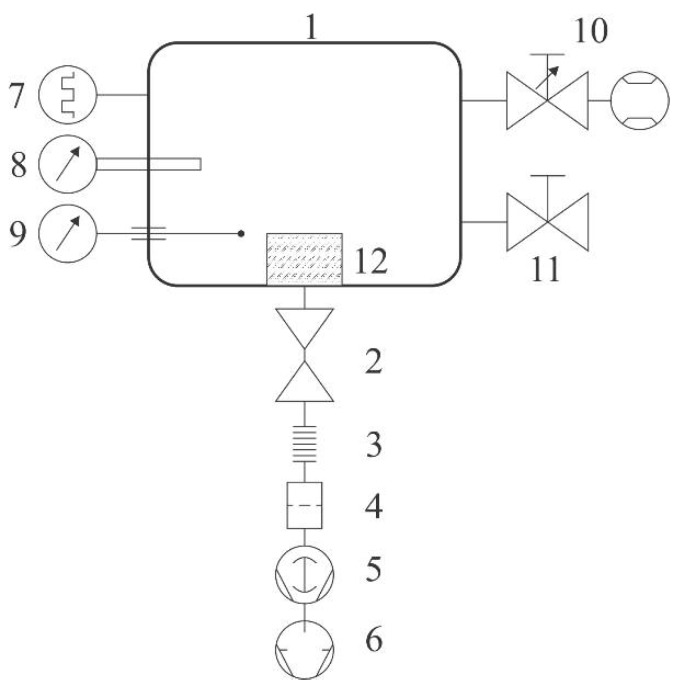
The schematic diagram of the vacuum of the small plasma reactor. 1—discharge chamber, 2—narrow tube with butterfly valve, 3—two-meter long bellows with diameter of 40 mm, 4—trap for fine powder, 5—Roots pump, 6—two-stage oil rotary pump, 7—Pirani vacuum gauge, 8—optical fiber with lens and spectrometer, 9—catalytic probe, 10—gas feeding flow measurement system, 11—air inlet valve, and 12—sample holder.

Samples of commercial polyethylene granules were treated in a plasma reactor (SurfaceTreat LA400, SurfaceTreat, a.s., Turnov, Czech Republic) suitable for the treatment of polymers on a semi-industrial scale. The PE granules were purchased from Sigma-Aldrich. The system’s schematic is shown in Figure 1. The discharge chamber of dimensions approx. 40 cm × 40 cm × 40 cm was made from aluminum. The native oxide layer forms on the aluminum surface, leading to rather inert properties against different plasmas of molecular gases, since the coefficient for heterogeneous surface recombination is low for various atoms [20]. The chamber had the volume of about 60 L and was pumped with a Roots pump backed by a two-stage rotary pump through a high efficiency particulate air (HEPA) filter, a butterfly valve, and bellows. The discharge chamber was equipped with a system for the gas inlet: an adjustable flow controller that allowed flows of up to 2 standard liters per minute. Two Microwave (MW) source was placed on the top flange of the plasma system. The source was forced-air cooled. The rest of the experimental system was kept at room temperature. Gaseous plasma of high luminosity was concentrated to the region a few centimeters thick next to the upper flange where the microwave source was mounted. The radiation was less intensive and rather homogeneous in the rest of the discharge chamber. Pressure inside the chamber was measured with a Pirani gauge calibrated for dry air. The pressure was adjusted by changing the gas flow through the flow controller, which was also calibrated for dry air, and/or by changing the tilt of the butterfly valve.

Plasma was characterized by optical emission spectroscopy (OES) and with a catalytic probe. An optical fibre was fixed to a window on a flange and connected to an optical spectrometer. We used an Avantes AvaSpec 3648 spectrometer (Avantes, Apeldoorn, The Netherlands). The device is based on the AvaBench 75 symmetrical Czerny Turner design, with a 3648-pixel charge-coupled device (CCD) detector and focal length of 75 mm. The range of measurable wavelengths is from 200 nm to 1100 nm, and the wavelength resolution is 0.5 nm. The spectrometer has a USB 2.0 interface, enabling high sampling rates of up to 270 spectra per second. It has a signal-to-noise ratio of 350:1. Integration time is adjustable from 10 µs to 10 min. At integration times of below 3.7 ms, the spectrometer itself performs internal averaging of spectra before transmitting them through the USB interface.

A catalytic probe for measuring the O-atom density was mounted at a flange and stretched 10 cm inside the discharge chamber in order to reveal the atom density away from the walls, where heterogeneous surface recombination might have influenced the probe signal. We used a professional laser-heated fiber-optics catalytic probe supplied by Plasmadis (Plasmadis Ltd., Ljubljana, Slovenia). The probe employed a catalytic tip made from pure cobalt with an oxide layer on the surface. The probe was sensitive to O atoms, but not to any N atoms that might be created in the gaseous plasma. For completeness of this paper, we shortly describe the main features as follows. The probe is made from a small catalytic tip, which is mounted onto an optical fibre. The other side of the fibre is connected to an electronic unit, which consists of an infrared (IR) radiation detector (for measuring tip temperature) and a laser (for heating the tip to elevated temperature). The electronic unit controls the laser power so that the tip temperature is held constant, irrespective from experimental conditions; in particular, the exothermic reactions that are likely to occur upon immersion of the probe tip into gaseous plasma. The flux of reactive plasma species onto the probe tip is then calculated from the difference in the laser power between plasma on and off conditions. After evacuation of the plasma reactor and before turning on the discharge, the catalytic tip was heated to a constant temperature (750 K, in this case) by the laser. When the discharge was turned on, the laser power dropped due to the extensive heterogeneous surface recombination of O atoms to parent molecules, and the electronic unit displayed the corresponding O-atom density in the vicinity of the tip. The details of the probe construction are described elsewhere [21]. The probe allows for rather precise measurements of the O-atom density, with the time resolution of less than a second and the start-up time of a few seconds. The absolute accuracy was about 20% and the relative accuracy about 3%, provided that the gas pressure does not change during the measurements.

Samples were placed onto a sample holder mounted at the bottom of the experimental system, as shown in Figure 1. The sample holder was in a form of a cup of diameter 20 cm and height 7 cm. A device for continuous mixing of granules upon plasma treatment was mounted onto the bottom of the cup. The granules filled the cup up to the level of about 1 cm from the bottom of the cup. A rather large diameter of granules (2 mm) allowed for efficient mixing, which in turn, allowed for the rather uniform treatment of the entire surface area of all the granules within a reasonable time. The treatment time was 1 min. The samples were commercially available polyethylene granules of diameter 2 mm. After evacuation of the discharge tube to the ultimate pressure, air was introduced into the discharge chamber through the flow controller upon continuous pumping so that a desired pressure was established. Different flow rates were adopted for this experiment. After the treatment, the plasma effects were studied by X-ray photoelectron spectroscopy (XPS).

We used a high-resolution instrument with monochromatized X-rays and a hemispherical electron analyzer (model TFA XPS Physical Electronics, Chanhassen, MN, USA), which employs monochromatic Al Kα_1,2_ radiation at 1486.6 eV for excitation of the surface over an area of about 400 µm^2^. Take-off angle was set at 45°. XPS survey-scan spectra were acquired at a pass-energy of 187 eV using an energy step of 0.4 eV. The high-resolution C1s spectra were acquired at a pass-energy of 23.5 eV using an energy step of 0.1 eV. The main C1s peak was fixed to a value of 284.8 eV for charge compensation. The XPS spectra were analysed using MultiPak v8.1c software (Ulvac-Phi Inc., Kanagawa, Japan, 2006) from Physical Electronics.

The morphology of granules was determined by an atomic force microscope. We used the instrument made by Solver PRO, NT-MDT, Moscow, Russia, which was operating in a tapping mode. The samples were scanned with a standard Si cantilever with a force constant of 22 N/m and at a resonance frequency of 325 kHz. The imaging was done on a 5 μm × 5 μm area of the samples.

## 3. Results and Discussion

**Figure 2:**
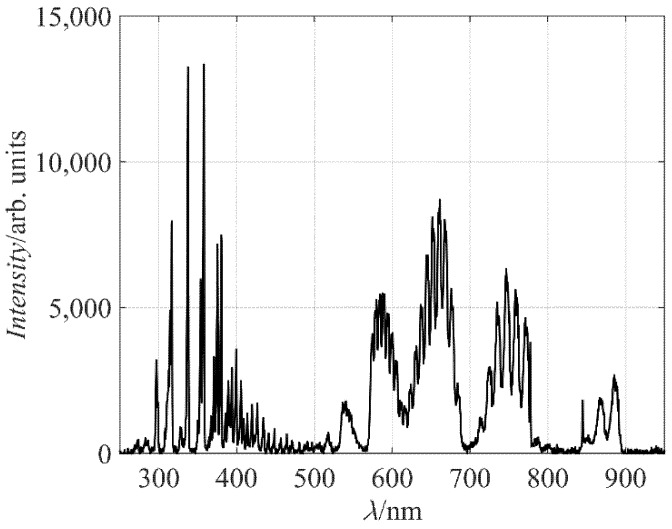
Optical emission spectrum of air plasma at the pressure of 80 Pa.

Figure 2 represents a typical spectrum of radiation arising from air plasma. The spectrum is rich in molecular bands. In the range of short wavelengths (roughly from 300 to 400 nm), there is a band corresponding to transitions between the C^3^Π_u_ to B^3^Π_g_ electronic states of a neutral nitrogen molecule. This radiation is not suppressed by any quantum rule, so it appears in a very short time after the state has been excited due to an electron impact. The potential energy of the upper state C^3^Π_u_ (ground vibrational level) is about 11 eV. Theoretically, the C^3^Π_u_ state can be created from the ground state of the nitrogen molecule (x^1^Σ^+^_g_). However, due to the large excitation energy of 11 eV, it is more likely that this state is created at an inelastic collision of an electron with a nitrogen molecule in a metastable state, most probably A^3^Σ^+^_u_, which has a radiative lifetime of 0.9 s [22].

The spectral range from about 500 to 900 nm is rich in another set of bands, arising from the relaxation of the B^3^Π_g_ to A^3^Σ^+^_u_ states. Numerous spectral features indicate rich population of vibrational states of both upper and lower electronic states. In fact, the most extensive “line” (broadened by rotational population) corresponds to the transition from the sixth vibrational level of the B^3^Π_g_ electronic state to the third vibrational level of the A^3^Σ^+^_u_ state. The excitation energy of the ground vibrational level of the B^3^Π_g_ state is just over 7 eV: about one-third of the excitation energy of the C^3^Π_u_ state. Also, the excitation cross section is large [23], so the intensity of the radiation arising from the B^3^Π_g_ to A^3^Σ^+^_u_ transitions is predominant in the spectrum shown in Figure 2.

Radiative transitions of atomic lines are barely visible, except from the oxygen line at 845 nm. The major oxygen line should be at 777 nm, but it overlaps with the nitrogen band in the spectrum, which was acquired by a rather low-esolution spectrometer. The excitation level of the O atoms radiating at 845 nm is about 11 eV: almost the same as the C^3^Π_u_ level of the nitrogen molecules. The reason for poor radiation from O atoms as compared to the C^3^Π_u_ to B^3^Π_g_ system is a smaller excitation cross section [24], rather than any difference in excitation energies, so the spectrum shown in Figure 2 only qualitatively shows the presence of neutral oxygen atoms in the plasma reactor. No radiation from excited N atoms is observed in Figure 2, although some N lines are positioned in the range of wavelengths not occupied by N_2_ transitions.

**Figure 3:**
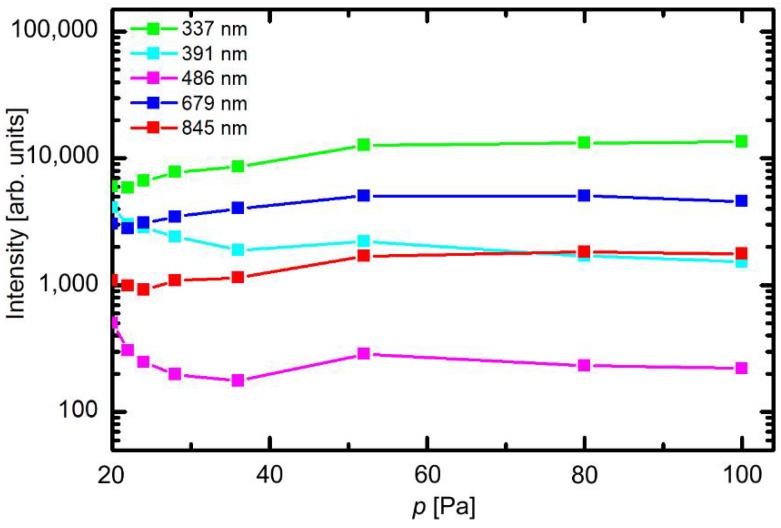
Intensity of selected spectral features versus pressure (*p*) in the discharge chamber.

The optical spectra were measured at different experimental conditions. The behavior of selected spectral features versus pressure at fully open butterfly valve (no pressure difference before or after the valve) is presented in Figure 3. The scale on the y-axis is logarithmic. The intensity of transitions within neutral nitrogen molecules (from the C^3^Π_u_ state at 337 nm and from the B^3^Π_g_ state at 679 nm) slowly decreases with decreasing pressure in the low-pressure range, while the intensity of positively charged molecular ions at 391 nm increases. This can be explained by a lower density of molecules and thus less frequent electron collisions; therefore, the electron temperature is somehow larger at lower pressures. This effect, however, is not pronounced, indicating that the plasma parameters do not change much with changing pressure. The atomic hydrogen line (H_β_ of the Balmer series) also increases with decreasing pressure for the same reasons. The emission from the neutral O atoms at 845 nm remains almost intact in the entire range of pressures.

**Figure 4:**
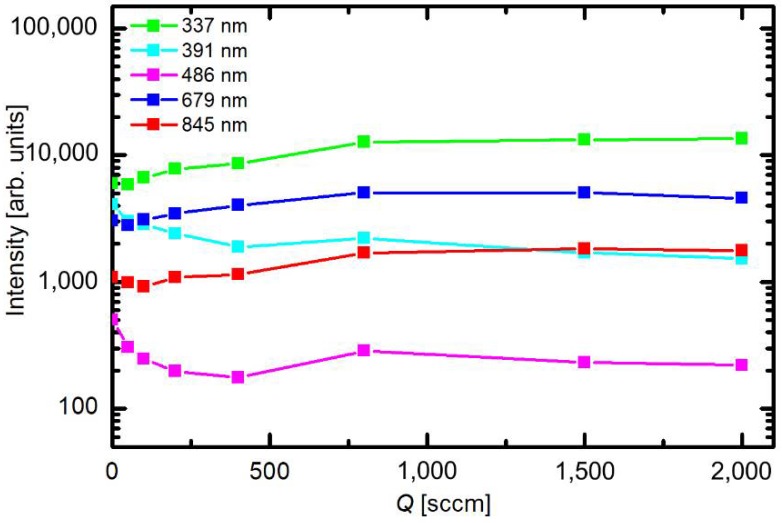
Intensity of selected spectral features versus air flow rate at the pressure of 80 Pa.

Figure 4 represents the variation of the spectral intensities versus gas flow at a constant pressure of 80 Pa. These set of experiments was performed by simultaneous change of the flow rate and opening of the butterfly valve. Figure 4 clearly shows that the flow rate does not influence the plasma parameters as long as the pressure remains constant. The resident time of gas inside the discharge chamber is obviously long enough that steady parameters are obtained irrespective of the gas drift through the discharge chamber. The optical spectra therefore indicate rather small variations of plasma parameters with pressure and almost constant parameters versus the flow rate, which make the system suitable for highly repeatable treatments of any samples mounted into the discharge chamber.

**Figure 5:**
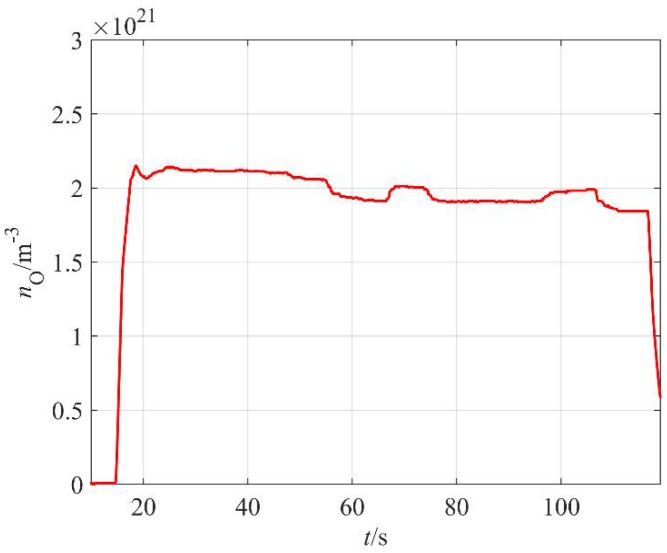
The behavior of O-atom density versus time (*t*) at 80 Pa and duty cycle 70%.

The absolute value of the O-atom density was measured with a catalytic probe. A typical probe signal versus time at constant discharge parameters is shown in Figure 5. As mentioned earlier, the start-up time of the commercial probe is several seconds. The probe shows rather constant O-atom density about half a minute after turning on the discharge, but a maximum is observed about 15 s after plasma ignition. The maximum is often explained by the presence of water vapor in the system; specifically, the fresh polymer granules contain some water, which is released from the surface upon plasma treatment. The water molecules are dissociated upon collisions with energetic gaseous species and contribute to the probe signal. The amount of adsorbed water is limited, and the vapor is continuously pumped away from the discharge chamber, so after about 30 s of plasma treatment, this contribution to the probe signal is marginal as compared to O atoms arising from the dissociation of oxygen molecules. A slow decrease in the O-atom density is observed for prolonged treatments and this could be due to thermal effects. It was already explained that the probability for heterogeneous surface recombination of atoms on surfaces of material facing plasma increases with increasing surface temperature [20]. The effect becomes important in the case of fibrous materials of poor thermal conductivity [25] and rather detrimental if more aggressive plasma is applied [26]. However, in our case, it remains marginal even for prolonged plasma treatment since in our case, the highly luminous (and thus reactive) plasma is concentrated to the region next to the MW sources, which are about 30 cm away from the sample holder.

**Figure 6:**
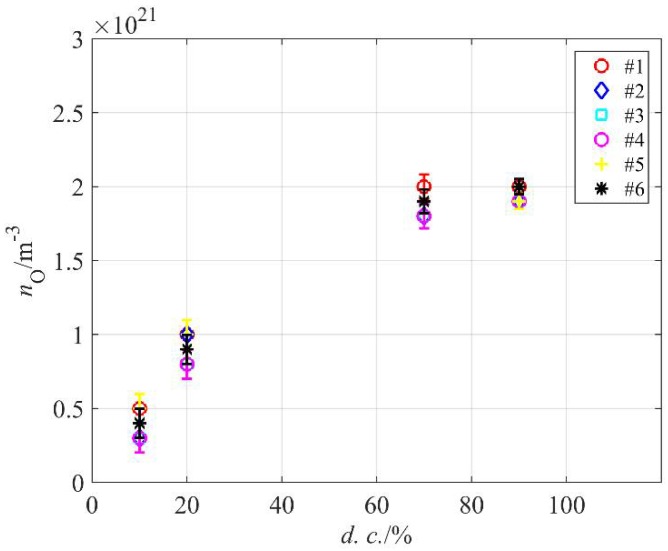
The O-atom density in air plasma at 80 Pa versus MW generator duty cycle.

The catalytic probe allowed for rather precise measurements of the O-atom density in our system. The reactor was loaded with PE granules and evacuated to ultimate pressure, and then air was introduced and the atom density was measured. This procedure was repeated six times in order to test and confirm repeatability. Figure 6 shows the density versus pressure at different duty cycles. As expected, the atom density is the highest at large duty cycles and decreases monotonously with decreasing cycles. At 10%, it is about four times smaller than at 100% or 80%. The best condition in terms of high atom density and energy efficiency is therefore at the duty cycle of about 70%.

**Figure 7:**
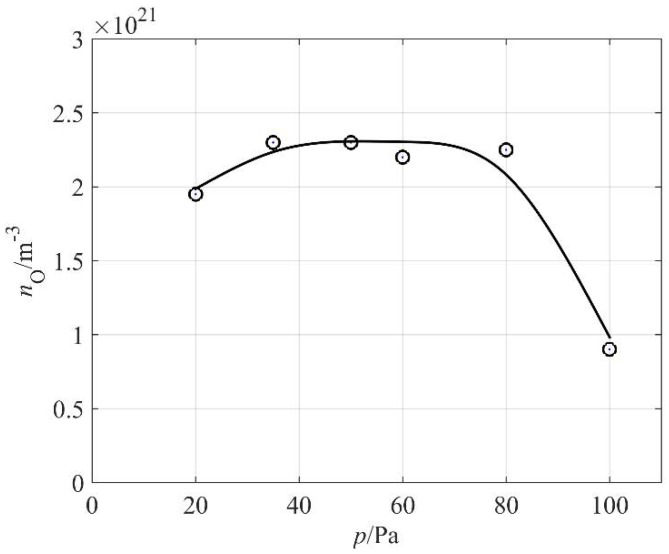
The O-atom density versus pressure at duty cycle 70%.

The O-atom density versus the pressure inside the discharge chamber is shown in Figure 7. One can observe a broad maximum: the atom density hardly changes in a range of pressures between 30 and 80 Pa. The absolute value is just over 2 × 10^21^ m^−3^, which is typical for plasma created in pure oxygen or a mixture of oxygen and argon [27,28,29]. The high density of O atoms in our weakly ionized plasma created in air can be attributed to differences in the excitation energies of oxygen and nitrogen molecules. As already mentioned, the first electronically excited level of a nitrogen molecule is A^3^Σ^+^_u_ at the potential energy of just over 6 eV. This state is the metastable state of a long radiative lifetime [22]. The dissociation energy of the oxygen molecule is somewhat lower at 5.2 eV. The quenching of the nitrogen metastable state by dissociative collision with an oxygen molecule is therefore energetically feasible. Furthermore, oxygen molecules exhibit two metastable electronic states at the potential energies of roughly 1 and 2 eV. The first metastable state, a^1^Δg, has a lifetime of almost an hour, so it is very stable at low pressure, at which the channels for relaxation are limited. Furthermore, according to Ionnin et al. [24], the cross section for excitation of the second state (b^1^Σ^+^_g_) from the metastable a^1^Δg state is rather large, even for electrons of kinetic energy 1 eV, and it peaks at a few eV, which is a typical electron temperature in low-pressure plasma [30]. Therefore, there are numerous channels for the dissociation of oxygen molecules in weakly ionized air plasma, which explains a rather high density of O atoms, as shown in Figure 7.

**Figure 8:**
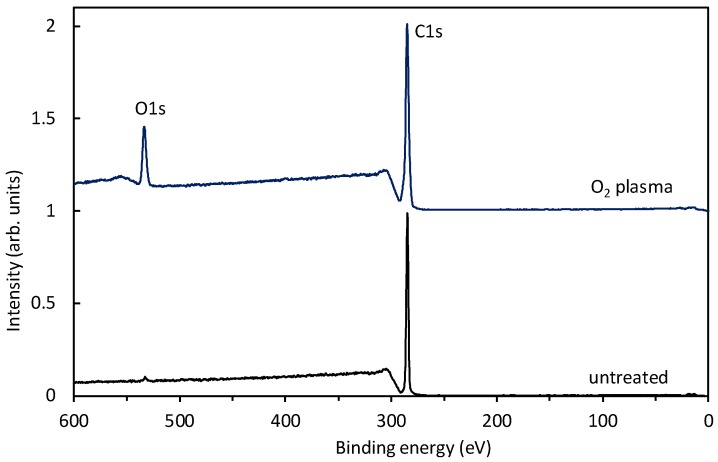
XPS survey spectra for an untreated and treated sample.

Samples of polyethylene granules were treated in air plasma and characterized by XPS soon after the treatment in order to limit any ageing effects. Specifically, the activation is not permanent, but hydrophobic recovery has been reported for numerous polymers with polar oxygen-rich functional groups on the surface [31]. Figure 8 shows survey spectra for an untreated sample and a sample treated for a minute at the pressure of 80 Pa, where the O-atom density was just over 2 × 10^21^ m^−3^ (see Figure 7). The survey spectrum for an untreated polyethylene shows only the carbon peak (which is sound with the polymer composition) and traces of oxygen. After the treatment, another peak appeared, indicating the incorporation of oxygen and an O-to-C ratio of 0.17.

Interestingly enough, no nitrogen peak is observed in Figure 8. Samples were treated at different pressures, and the survey spectra were practically identical to those of Figure 8. The absence of nitrogen on the polymer surface is attributed to a higher affinity for reactions with O atoms and also to the shortage of nitrogen atoms in the plasma reactor. As revealed from Figure 2, plasma cannot be rich in N atoms, because no peak is observed. The very low concentration of N atoms as compared to O atoms can be explained by numerous reasons. The first one is the high dissociation energy of N_2_ molecules, which is about 10 eV (twice that of the value for oxygen molecules). The plasma electrons are therefore unlike to dissociate a nitrogen molecule in the ground electronic state, even if vibrationally excited. Nitrogen atoms could be created by dissociation of the metastable nitrogen molecule in the A^3^Σ^+^_u_ electronic state. This state is the final state of the neutral molecule emission bands observed in Figure 2. Furthermore, the A^3^Σ^+^_u_ electronic state is also well populated with vibrational states, since many radiative transitions occur to higher vibrational levels [32]. On the other hand, this electronically excited state is quenched by reactions with other plasma species as explained above, thus its density is probably much lower than the O-atom density in our plasma reactor. In fact, Ricard et al. [33] measured the density of the A^3^Σ^+^_u_ electronic state in a similar plasma system and observed values of the order of 10^17^ m^−3^, which is four orders of magnitude smaller than the O-atom density in our experimental setup. The density of N atoms is therefore orders of magnitude smaller than the density of O atoms, which together with other considerations, explains the absence of an N peak in Figure 8.

**Figure 9:**
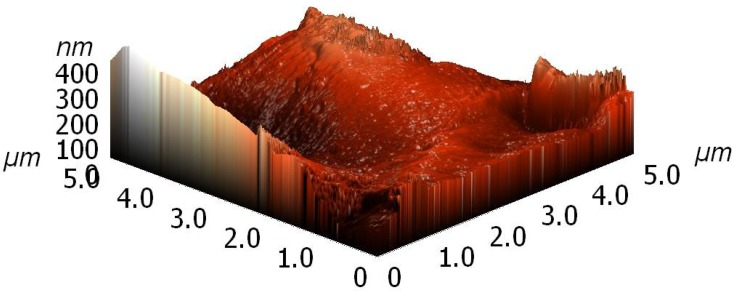
Three-dimensional atomic force microscopy (AFM) image of a 5 μm × 5 μm area of an untreated granule.

Both untreated and plasma treated samples were characterized by atomic force microscopy (AFM). A typical image is shown in Figure 9. Images were obtained at various spots on a granule and they differed significantly. The as-produced granules were therefore of rich morphology. After plasma treatment, no significant deviation was observed, but it should be stressed again that the granules were already rough before the treatment, so any change in topography that could be a result of plasma treatment was difficult to detect.

**Figure 10:**
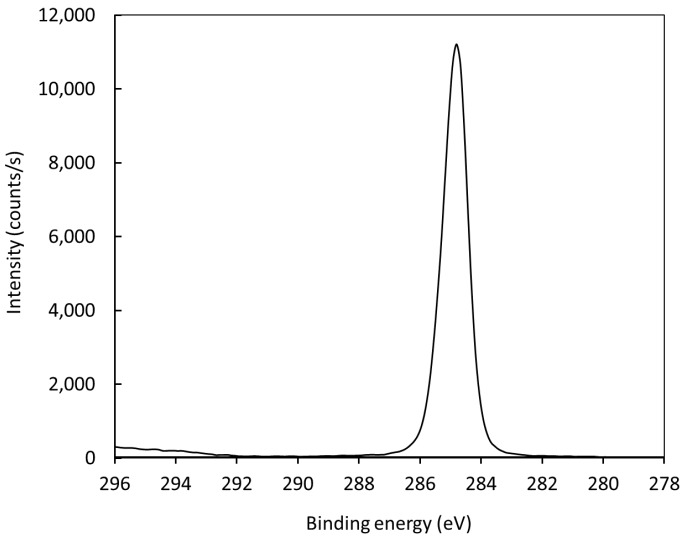
High-resolution C1s spectrum for an untreated sample.

**Figure 11:**
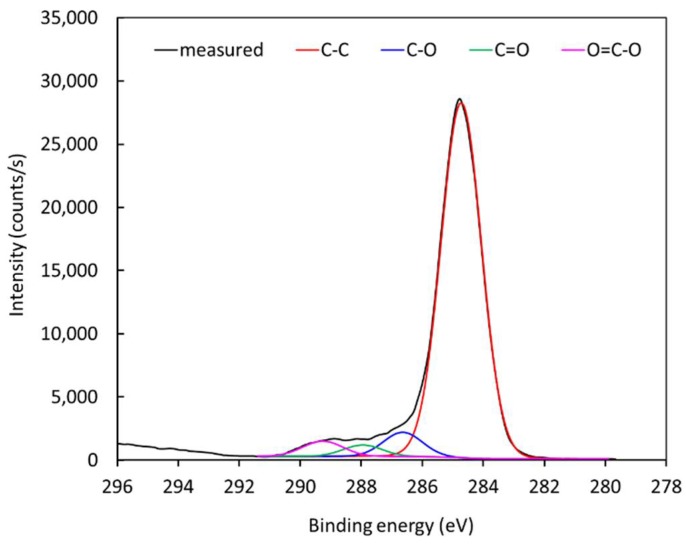
High-resolution C1s spectrum for a treated sample.

Figure 10 represents the high-resolution C1s peak of the photoelectron spectrum for an untreated polyethylene sample. The peak is almost perfectly symmetrical, indicating no functionalization of the polymer surface with groups that cause asymmetrical peaks. After treatment with air plasma, the peak becomes asymmetrical due to surface oxidation. The C1s peak for a treated polymer sample is shown in Figure 11. Since Figure 8 shows negligible (if any) concentration of other elements, it is feasible to deconvolute the peak in Figure 11 with oxygen-containing functional groups. The best fit was obtained using only hydroxyl, carbonyl, and carboxyl/ester groups. The subpeaks corresponding to these functional groups are shown in Figure 11. The result was quantified using the software package and revealed the following concentrations of groups: C–C 88.5%, C–O 5.4%, C=O 2.6%, and O=C–O 3.5%. The fit is almost perfect at the high-energy tail, indicating that carboxyl/ether groups are those of the highest energy shift. Some authors have also observed a carbonate peak on plasma-treated polymers, but such functional groups obviously have not appeared on polyethylene in our case [34,35,36,37,38,39].

Our results for activation of polyethylene granules are similar to those for the same material, but of different morphology. Lopez-Santo [15] employed a remote MW source for the treatment of originally smooth PE, but using an argon–oxygen gas mixture, and observed the same O/C ratio (0.2) as we did in the case of the granules. Abou Rich et al. [40] employed Ar/O_2_ and HeO_2_ mixtures using a completely different discharge (plasma torches) and observed the much higher O/C ratio of 0.36, irrespective from the gas mixture. Pandiyaraj et al. [41] used a simple direct current (DC) discharge in different gases and observed the highest O/C ratio using air: it was up to twice as high as in the case of pure oxygen plasma. Bílek et al. [42] observed a much lower O/C ratio after treatment of PE in air plasma, but provided no details about the discharge system. Sanchis [43] and Vesel [44] used radiofrequency discharges to functionalize polyethylene in oxygen plasma and observed quick saturation of the PE surface with oxygen groups. The O/C ratio depended on the coupling of the radiofrequency generator: higher values were observed for electrodeless coupling using a coil than for standard capacitive coupling with electrodes inside the discharge chamber. In contrary to the above works, Borcia et al. [45] obtained a O/C ratio as high as 0.68, but they used an atmospheric pressure discharge in air, which may not be suitable for the uniform treatment of large quantities of PE granules.

## 4. Conclusions

Thorough characterization of the reactor employing a pulsed microwave discharge for sustaining plasma in a metallic chamber revealed interesting properties, which are extremely suitable for the functionalization of polyethylene granules. The plasma parameters do not depend much on the pressure in the discharge chamber and are practically independent from the flow of gas through the reactor. The O-atom density exhibited a broad maximum in the range of pressures between about 30 Pa and 80 Pa, so the surface functionalization with polar oxygen-rich functional groups can be accomplished in a highly reproducible manner even if the pressure slightly varies from batch to batch. The absolute value of the O-atom density is about 2 × 10^21^ m^−3^, which assures for rapid functionalization of the polypropylene granules. The XPS spectra revealed a negligible concentration of nitrogen on the polymer surface, which was explained by a small density of N atoms in the plasma reactor as well as preferential interaction with O atoms. The plasma treatment resulted in functionalization with hydroxyl, carbonyl, and carboxyl/ester groups.

## References

[1] Vesel A., Mozetic M. (2017). New developments in surface functionalization of polymers using controlled plasma treatments. J. Phys. D Appl. Phys..

[2] Gorjanc M., Mozetič M. (2014). Modification of Fibrous Polymers by Gaseous Plasma: Principles, Techniques and Applications.

[3] Vukušić T., Vesel A., Holc M., Ščetar M., Jambrak A.R., Mozetič M. (2018). Modification of physico-chemical properties of acryl-coated polypropylene foils for food packaging by reactive particles from oxygen plasma. Materials.

[4] Orendáč M., Čižmár E., Kažiková V., Orendáčová A., Řezníčková A., Kolská Z., Švorčík V. (2018). Radicals mediated magnetism in Ar plasma treated high-density polyethylene. J. Magn. Magn. Mater..

[5] Vartiainen J., Pasanen S., Kentt E., Vähä-Nissi M. (2018). Mechanical recycling of nanocellulose containing multilayer packaging films. J. Appl. Polym. Sci..

[6] Zhao Z.P., Zhang A.S., Wang X.L., Lu P., Ma H.Y. (2018). Controllable modification of polymer membranes by LDDLT plasma flow: Grafting acidic ILs into PPF membrane for catalytic performance. J. Membr. Sci..

[7] Hu Z., Chng S., Liu Y., Moloney M.G., Parker E.M., Wu L.Y.L. (2018). One-step chemical functionalization of polyethylene surfaces via diarylcarbene insertion. Mater. Lett..

[8] Ozaltin K., Lehocky M., Humpolicek P., Vesela D., Mozetic M., Novak I., Saha P. (2018). Preparation of active antibacterial biomaterials based on sparfloxacin, enrofloxacin, and lomefloxacin deposited on polyethylene. J. Appl. Polym. Sci..

[9] Muzammil I., Li Y.P., Li X.Y., Lei M.K. (2018). Duty cycle dependent chemical structure and wettability of RF pulsed plasma copolymers of acrylic acid and octafluorocyclobutane. Appl. Surf. Sci..

[10] Van Vrekhem S., Vloebergh K., Asadian M., Vercruysse C., Declercq H., Van Tongel A., De Wilde L., De Geyter N., Morent R. (2018). Improving the surface properties of an UHMWPE shoulder implant with an atmospheric pressure plasma jet. Sci. Rep..

[11] Lindner M., Rodler N., Jesdinszki M., Schmid M., Sängerlaub S. (2018). Surface energy of corona treated PP, PE and PET films, its alteration as function of storage time and the effect of various corona dosages on their bond strength after lamination. J. Appl. Polym. Sci..

[12] Popelka A., Khanam P.N., Almaadeed M.A. (2018). Surface modification of polyethylene/graphene composite using corona discharge. J. Phys. D Appl. Phys..

[13] Popelka A., Novák I., Al-Maadeed M.A.S.A., Ouederni M., Krupa I. (2018). Effect of corona treatment on adhesion enhancement of LLDPE. Surf. Coat. Technol..

[14] López-Santos C., Yubero F., Cotrino J., González-Elipe A.R. (2011). Nitrogen plasma functionalization of low density polyethylene. Surf. Coat. Technol..

[15] López-Santos C., Yubero F., Cotrino J., Barranco A., Gonzlez-Elipe A.R. (2008). Plasmas and atom beam activation of the surface of polymers. J. Phys. D Appl. Phys..

[16] López-García J., Bílek F., Lehocký M., Junkar I., Mozetič M., Sowe M. (2013). Enhanced printability of polyethylene through air plasma treatment. Vacuum.

[17] Oberbossel G., Güntner A.T., Kündig L., Roth C., Von Rohr P.R. (2015). Polymer powder treatment in atmospheric pressure plasma circulating fluidized bed reactor. Plasma Process. Polym..

[18] Inagaki N., Tasaka S., Abe H. (1992). Surface modification of polyethylene powder using plasma reactor with fluidized bed. J. Appl. Polym. Sci..

[19] Park S.H., Kim S.D. (1994). Plasma surface treatment of HDPE powder in a fluidized bed reactor. Polym. Bull..

[20] Mozetič M., Primc G., Vesel A., Zaplotnik R., Modic M., Junkar I., Recek N., Klanjšek-Gunde M., Guhy L., Sunkara M.K. (2015). Application of extremely non-equilibrium plasmas in the processing of nano and biomedical materials. Plasma Sources Sci. Technol..

[21] Primc G., Mozetič M., Cvelbar U., Vesel A. (2015). Method and Device for Detection and Measuring the Density of Neutral Atoms of Hydrogen, Oxygen or Nitrogen. WIPO Patent.

[22] Zipf E.C. (1963). Measurement of the Diffusion Coefficient and Radiative Lifetime of Nitrogen Molecules in the A3Σ_u_^+^ State. J. Chem. Phys..

[23] Itikawa Y. (2006). Cross sections for electron collisions with nitrogen molecules. J. Phys. Chem. Ref. Data.

[24] Ionin A.A., Kochetov I.V., Napartovich A.P., Yuryshev N.N. (2007). Physics and engineering of singlet delta oxygen production in low-temperature plasma. J. Phys. D Appl. Phys..

[25] Gorjanc M., Mozetič M., Vesel A., Zaplotnik R. (2018). Natural dyeing and UV protection of plasma treated cotton. Eur. Phys. J. D.

[26] Gorjanc M., Mozetič M., Primc G., Vesel A., Spasić K., Puač N., Petrović Z.L., Kert M. (2017). Plasma treated polyethylene terephthalate for increased embedment of UV-responsive microcapsules. Appl. Surf. Sci..

[27] Altaweel A., Imam A., Ghanbaja J., Mangin D., Miska P., Gries T., Belmonte T. (2017). Fast synthesis of ultrathin ZnO nanowires by oxidation of Cu/Zn stacks in low-pressure afterglow. Nanotechnology.

[28] Kutasi K., Noël C., Belmonte T., Guerra V. (2016). Tuning the afterglow plasma composition in Ar/N_2_/O_2_ mixtures: Characteristics of a flowing surface-wave microwave discharge system. Plasma Sources Sci. Technol..

[29] Gueye M., Gries T., Noël C., Migot-Choux S., Bulou S., Lecoq E., Choquet P., Belmonte T. (2016). Interaction of (3-Aminopropyl)triethoxysilane With Late Ar-N_2_ Afterglow: Application to Nanoparticles Synthesis. Plasma Process. Polym..

[30] Gibson A.R., Foucher M., Marinov D., Chabert P., Gans T., Kushner M.J., Booth J.P. (2017). The role of thermal energy accommodation and atomic recombination probabilities in low pressure oxygen plasmas. Plasma Phys. Control. Fusion.

[31] Praveen K.M., Thomas S., Grohens Y., Mozetič M., Junkar I., Primc G., Gorjanc M. (2016). Investigations of plasma induced effects on the surface properties of lignocellulosic natural coir fibres. Appl. Surf. Sci..

[32] Pashov A., Popov P., Knöckel H., Tiemann E. (2008). Spectroscopy of the a^3^∑_u_ + state and the coupling to the X^1^∑_g_ + state of K_2_. Eur. Phys. J. D.

[33] Ricard A., Oh S., Jang J., Kim Y.K. (2015). Quantitative evaluation of the densities of active species of N_2_ in the afterglow of Ar-embedded N_2_ RF plasma. Curr. Appl. Phys..

[34] Quoc Toan Le Q.T., Pireaux J.J., Caudano R. (1997). XPS study of the PET film surface modified by CO_2_ plasma: Effects of the plasma parameters and ageing. J. Adhes. Sci. Technol..

[35] Arefi F., Andre V., Montazer-Rahmati P., Amouroux J. (1992). Plasma polymerization and surface treatment of polymers. Pure Appl. Chem..

[36] Vesel A., Mozetic M., Hladnik A., Dolenc J., Zule J., Milosevic S., Krstulovic N., Klanjek-Gunde M., Hauptmann N. (2007). Modification of ink-jet paper by oxygen-plasma treatment. J. Phys. D Appl. Phys..

[37] O’Hare L.A., Leadley S., Parbhoo B. (2002). Surface physicochemistry of corona-discharge-treated polypropylene film. Surf. Interface Anal..

[38] Kregar Z., Bišćan M., Miloševiá S., Mozetič M., Vesel A. (2012). Interaction of argon, hydrogen and oxygen plasma early afterglow with polyvinyl chloride(PVC) materials. Plasma Process. Polym..

[39] Zaldivar R., Nokes J., Patel D.N., Morgan B.A., Steckel G., Kim H.I. (2012). Effect of using oxygen, carbon dioxide, and carbon monoxide as active gases in the atmospheric plasma treatment of fiber-reinforced polycyanurate composites. J. Appl. Polym. Sci..

[40] Abou Rich S., Dufour T., Leroy P., Reniers F., Nittler L., Pireaux J.J. (2015). LDPE surface modifications induced by atmospheric plasma torches with linear and showerhead configurations. Plasma Process. Polym..

[41] Pandiyaraj K.N., Deshmukh R.R., Ruzybayev I., Shah S.I., Su P.G., Halleluyah M., Halim A.S. (2014). Influence of non-thermal plasma forming gases on improvement of surface properties of low density polyethylene (LDPE). Appl. Surf. Sci..

[42] Bílek F., Křížová T., Lehocký M. (2011). Preparation of active antibacterial LDPE surface through multistep physicochemical approach: I. Allylamine grafting, attachment of antibacterial agent and antibacterial activity assessment. Colloids Surf. B Biointerfaces.

[43] Sanchis R., Fenollar O., García D., Sánchez L., Balart R. (2008). Improved adhesion of LDPE films to polyolefin foams for automotive industry using low-pressure plasma. Int. J. Adhes. Adhes..

[44] Vesel A. (2008). XPS study of surface modification of different polymer materials by oxygen plasma treatment. Inf. MIDEM.

[45] Borcia G., Anderson C.A., Brown N.M.D. (2004). The surface oxidation of selected polymers using an atmospheric pressure air dielectric barrier discharge. Part I. Appl. Surf. Sci..

